# On Acoustic Voice Quality Index measurement reliability in digital health applications: a narrative review and empirical evaluation of speech sample length requirements

**DOI:** 10.3389/fdgth.2025.1610353

**Published:** 2025-11-24

**Authors:** Fredrik Nylén

**Affiliations:** Department of Clinical Sciences, Division of Speech and Language Pathology, Umeå University, Umeå, Sweden

**Keywords:** AVQI, reliability, dysphonia assessment, simulation study, narrative review

## Abstract

The Acoustic Voice Quality Index (AVQI) is a widely adopted tool for assessing dysphonia, incorporating sustained vowel and continuous speech samples to enhance ecological validity. Despite its broad use, the reliability of AVQI measurements, particularly in digital health applications, remains underexplored. This study aims to review the literature on AVQI's development and validation from the perspective of internal consistency of acoustic measurements, and to assess the amount of speech required to reliably determine the AVQI of a voice recording. Two sub-studies are described. Study 1: A narrative review was conducted using Google Scholar and Scopus to identify studies mentioning “AVQI” and “reliability”. Key methodological details were extracted from studies reporting AVQI measurements, summarized, and discussed in terms of how the internal consistency of acoustic measurements was ensured across studies where AVQI had been validated or applied. Study 2: Recordings of read and spontaneous speech as well as sustained vowels produced by 54 native Swedish speakers (22 female, 32 male; age range: 46–78 years) were assessed in terms of the amount of speech required to obtain a reliable acoustic measurement of the speakers' voices. Simulations were performed using read and spontaneous speech materials of varying lengths. The variability in AVQI and its sub-measures was analyzed relative to the length (in words or in seconds) of continuous speech included. The result of study 1 shows that out of 129 identified studies, 85 reported on AVQI measurements. The review highlighted substantial variability in continuous speech lengths used across studies, ranging from 4–200 words. A recommendation of 3 s of voiced segments was often adhered to, but was found to lack sufficiently robust grounding. The simulations indicated that AVQI achieves internal consistency at speech lengths of approximately 50 words (or 20 s), which is longer than the current recommendation. Both read and spontaneous speech provided stable AVQI measurements at these speech lengths. AVQI thresholds obtained using speech lengths shorter than 50 words (20 s) may require re-evaluation. Robust standardization of continuous speech lengths is essential for the successful adoption of AVQI in digital health applications.

## Introduction

The ability to screen for symptoms of dysphonia hinges on the reliability of the methods and the ability to replicate the observation outside of the screening context. Acoustic analysis is well recognized as a promising domain for developing diagnostic tools for digital health applications for dysphonia, due to its high availability and relatively moderate resource requirements. The simplicity of the data acquisition procedure, often requiring only a microphone of sufficient quality and a quiet room to perform, allows administration of non-expert clinical staff to document voice symptoms, as well as experts to support symptom diagnosis. Many methods for screening and diagnostics based on a single or a few acoustic properties have been proposed. While these methods can be successful in making simple distinctions about whether a voice is likely to have dysphonia, some lack the ecological validity needed for comprehensive assessment. Sustained vowel production remains the cornerstone of voice quality assessment due to the affordance for assessing properties of phonation and respiration in an environment free from interference caused by dynamics in laryngeal and articulatory muscles.

The Acoustic Voice Quality Index (AVQI) is a digital health assessment and screening method for voice impairment that was designed to achieve a higher ecological validity over then existing assessment techniques by also incorporating continuous speech into the analysis (1, 2). The AVQI has been shown to have a high validity in terms of its ability to correctly identify a range of vocal fold mass lesions (vocal fold polyps, Reinke's hyperplasia, carcinoma, chronic hyperplastic laryngitis with keratosis, papillomata, vocal fold nodules, keratosis without laryngitis, and amyloid), nonorganic voice disorders (ventricular and functional dysphonias), other benign voice disorders (laryngeal cysts, sulcus glottidis, laryngeal web, arytenoid granuloma), and voice disorders of neurological diseases (vocal fold paralysis, Parkinson's disease, and Huntington's disease) (2). The AVQI incorporates a linear combination of acoustic voice measures (the cepstral peak prominence (CPPS), Harmonic to Noise Ratio (HNR), the Slope and Tilt of the Long Time Averaged Spectrum (LTAS Slope and LTAS Tilt), the local absolute Shimmer (Shim local), and dB scaled local Shimmer (Shim local DB)) into a single index (1.0–10.0). The AVQI is sensitive to language-specific features and requires a local validation to serve as a point of reference in the evaluation. Local validation efforts determine the threshold value for separating voices produced by normophonic (without impairment) and dysphonic speakers. A recent systematic review concluded that the language-specific AVQI thresholds ranges between 1.33 and 3.15 for the most recent version of the AVQI implementation (3). An AVQI change of 0.95 has been found to reflect the observation of a clinically meaningful change by a clinical expert in formal assessment of symptom worsening or improvement (4). A high likelihood of a clinically meaningful change in a patients self-perceived level of disability is suggested by an AVQI change of 1.36 or more. The AVQI has accessible computer implementations (5, 6) and efforts are currently being made to implement the AVQI in smartphones to increase clinical availability (7).

Successful adoption of digital health assessment or screening hinges on the psychometric properties of the entire measurement and evaluation procedure (8). The neglect of reliability issues in digital health information is, however, a well-recognized problem (9, 10). In acoustic assessment of voices, outcomes are well recognized to be sensitive to the influence of several factors, such as recording conditions and the recording equipment's technical specification and positioning (11–15). When dynamic speech audio signals form the basis for the assessment, the outcome is also susceptible to influence by the introduction of a continuously changing nature of the voice signal. If an assessment procedure is likely to change depending on which exact portion of speech was injected, the internal consistency (16) is considered at risk and must be managed. Ensuring robustness against these sources of influence, either in the algorithm or through safeguards aimed at ensuring the equivalence of materials at data acquisition, is therefore essential. Failing to do so may compromise the outcome and raise questions about the method's validity in clinical practice.

The AVQI is a well-researched assessment procedure that has been concluded to afford detection of dysphonia and estimation of dysphonia severity across many languages. A cursory view of reports, however, reveals that there may be substantial variation in how much continuous speech material the AVQI analysis was based on across studies. Prominent validation studies use approximately 3 s (17), a small number of words in the 12–15 approximate range (18), 50–70 words (19, 20), or substantially longer sequences (21). Varying material bases across validations of the measure is, in itself, not a significant issue, provided that the material is well-suited to form the basis for an assessment of the speaker's voice quality. However, with the observation of widely different materials being used across validation studies, the degree to which material selection and its impact on the reliability and ensured validity of the AVQI demands a more thorough investigation. Further, it becomes of interest to investigate the speech material length required to afford assessment of AVQI with good internal consistency, so that it can be relied on to capture the presence and severity of dysphonia in a person's voice.

The aims of this study were to 1) perform a narrative review of studies in which the AVQI is validated or used from viewpoint of how reliability of the acoustic measurements was ensured or investigated, and 2) determine through simulation how much continuous speech material is likely to be required to provide AVQI measurements with good internal consistency in outcomes, both in read and spontaneous speech.

## Study 1—a narrative review of the adoption of reliability safeguards for the AVQI

The aim of Study 1 was to provide an evidence-informed (22) view of the validation of the AVQI, and how it has been used in published studies to assess the level of voice impairment in a clinical population. A narrative review using a broad search strategy was performed, but with the specific aim to capture methodological aspects related to how the reliability, internal consistency, and validity of the AVQI have been ensured and estimated.

### Methods

#### Materials

A broad search for “reliability” in combination with “AVQI” was performed in Google Scholar and Scopus on March 11th, 2025. The exact query used was “[ALL (‘reliability’) AND ALL (‘AVQI’)]”, and no date limits were applied. The inclusion criteria were 1) that the study was an original report of an empirical investigation published either in a peer-reviewed journal or in conference proceedings, 2) the study should provide information from AVQI measurements explicitly made to meet the reviewed study's aims, 3) that sufficient methodological details were made available to determine the nature of the material the AVQI was based on, and 4) that the required methodological details were made available in English, Swedish, German or Spanish so that the author could confidently extract them. Correspondingly, studies were excluded from the extraction of information of interest if the AVQI was only mentioned or described and not used for empirical measurement for the specific report, or if the methodological details were insufficient or unavailable to the author due to language constraints.

From each study included in the analysis, information on the language being spoken by the speakers, publication year, the number of participants, the length of the sustained vowel sample, the length of continuous speech included, the nature of the task the speakers were asked to perform to collect the continuous speech, and aspects of the method that aimed to assess the reliability of the acoustic measurement of AVQI was collected. When possible, in relation to the availability of language-specific standardized texts, reported text lengths, such as the number of text initial syllables or sentence numbers in the text, were converted to the corresponding number of words. When the standard text could not be obtained, the original reported quantity was retained in the summary table. While not the primary focus of the review, the study outcomes were also compiled for each study. Further, the studies were placed in broad categories of Validation studies, Reliability assessment studies, and Application studies, with an allowance for one study to belong to more than one category.

All identified articles were analysed by the author using a standardized screening form. To investigate the inter-rater reliability of the information extraction, a repeated analysis was performed on a random selection of 20 articles for inclusion/exclusion decisions, study classification (Validation study, Reliability assessment study, and Application study), number of participants included, and whether information on an effort to assess or ensure measurement reliability was reported.

#### Results

In total, 129 studies mentioning “AVQI” and “reliability” were identified. 16 studies discussed AVQI measures that were not collected for the study or referred to material published elsewhere in a local language. 28 studies mentioned the AVQI in passing without using the measure in the study, or did not provide detailed information on how the measurements had been performed, and could not form a basis for reliability assessment. 85 studies (1–5, 17–21, 23–95) included information on the materials used and how the AVQI had been obtained, and were subsequently included in the analysis. For two studies, only partial information could be extracted. The repeated analysis of a random selection of 20 articles showed a 91% agreement with the original determination of inclusion/exclusion decisions, study classification (Validation study, Reliability assessment study, and Application study), number of participants included, and whether information on an effort to assess or ensure measurement reliability was reported. Discrepancies between analyses were resolved, and the final information extracted from the included studies is presented in Supplementary Materials A (1–5, 17–21, 23–95).

The AVQI measure is computed from two types of materials, a sustained vowel and a section of continuous speech. With regard to the sustained vowel, there is a rudimentary agreement among studies that a vowel sample of at least 2 s should be collected, and most studies include a 3 s sustained vowel sample. The middle portion was primarily where the vowel sample was extracted, but some studies considered instead the stability of the vowel production when determining the portion to extract. In local contexts, it was common to give the speaker more than one production attempt and then use the most stable or otherwise suitable production. The use of the entire vowel, or an unspecified part of it, was not uncommon across studies, and some studies reported an aim of collecting a vowel that was just not too short to be their primary methodological consideration.

For the continuous speech portion, a near consensus of asking the participant to read a text out loud was observed among studies. Localized use of a verbal task where the participant is asked to count from one to ten is also observed. No identified study used spontaneous speech. A wide range of lengths of texts (4–200 words) that the participants were asked to read is observed. A recommendation by key investigators of the AVQI (23) of using 3 s of voiced portions of speech was identified, and was often, but not exclusively, adhered to. The recommendation was made based on an evaluation of speech sample lengths in three categories, where the other lengths were a 12 word portion or the full 60-word standard text, respectively.

Two studies report the duration (in seconds) of samples (25); the number of sentences, words, or syllables was, however, observed to be the dominant unit used when reporting continuous speech lengths. The impact of the length of continuous speech material is considered only in one study (23). The length of material used when validating the AVQI in a local context is not consistently adhered to in subsequent applied studies in the same language.

While perceptual or clinical rater reliability is assessed and reported in applicable studies, the corresponding attention to assessing or ensuring the degree of acoustic measurement reliability is not frequently observed. The study by Barsties et al. (96) is a notable exception, where portions of different lengths of the read text were evaluated, where the 3 s material used achieved overall improved performance in the 0.03–0.08 range across performance metrics over the 12 and 60-word text length alternatives. One study assessed whether manual identification of voiced segments is required, with the conclusion that a standardized length of 27 syllables provided comparable results whether manually segmented or not (54), which may be up to 38 syllables in other languages (96).

Comparisons of different versions of AVQI are made within the same acoustic material, and estimated sensitivity, specificity, accuracy, or area under the receiver operating characteristic curve (ROC AUC) are performed in the entire data sample. In only one validation study (77), a separate evaluation set for determining sensitivity, specificity, and ROC AUC of their proposed AVQI threshold value for distinguishing homophonic and dysphonic speakers was used. Balanced groups of dysphonic and normophonic speakers are not generally observed in the reviewed studies. Maryn et al. (1) performed cross-validation for the correlation of AVQI with the perceptual evaluation, but not when deriving the AVQI threshold or when computing performance measures (sensitivity, specificity, ROC AUC) for the AVQI model. In relation to the risk of AVQI thresholds being selected based on a model that is overfitted to the data it was trained on and not transferable to new materials with retained predictive accuracy (96, 97), only the Lithuanian language has a validation for which the sensitivity and specificity obtained under a reliable statistical methodology (77). The reported cross-validated performance reported by Maskeliūnas et al. (77) is noted to be lower than the performances reported in other, not cross-validated, validation studies.

A methodological issue raised by the review is the use of lossy compression algorithms (MP3 encoding) when storing the acoustic recording before AVQI analysis. One study (38) report storage of speech recordings in the MP3 format. A complementary search of “AVQI” and “MP3” anywhere in a report [ALL (“MP3”) AND ALL (“AVQI”)] found one other study using this procedure for their recordings while computing the AVQI (98). A second study (39), excluded from the initial review set due to AVQI only being discussed and never applied, discussed in general terms that MP3 format may be safe to use for spectrally localized measurements, such as fundamental frequency, while measures influenced by more of the spectrum may be more sensitive to the destructive compression used, citing an earlier study (100) as the basis for drawing this conclusion. While the conclusion that fundamental frequency may be robust to lossy compression has been given additional support in more recent works (101) the CPP and LTAS subcomponents of AVQI, for instance, may be less robustly reproduced when the storage file format is not considered (102). However, since lossy compression formats (like the MP3 format) were not extensively used in the reviewed literature, the issue was not considered further in the review.

#### Discussion of study 1 findings

The results of the narrative review revealed a substantial variability in continuous speech lengths used (4–200 words) in the validations of AVQI across languages. Since AVQI is evaluated against a threshold determined for each language, the impact of variability in source materials is not problematic, provided that AVQI measurements are compared against comparable results in the local validation. However, the narrative review also noted a concerning trend of applied studies not always following the data application procedure of the validation study for the local language. A meta-analysis of AVQI threshold values found in validation studies for different languages has been made available (3), but requires updating to include the many validation efforts reported on since its publication. The robustness of a commonly cited recommendation of 3 s of voiced segments, which is frequently adhered to in studies, may be questioned on methodological grounds, and demands external validation.

Methods for achieving a good balance between the ecological validity of AVQI outcomes and the internal consistency of the measure demand increased attention. In recordings of continuous speech, the acoustic signal is constantly changing, and continuous speech was incorporated in the AVQI procedure with the aim of increasing the ecological validity of assessments (1). The recommendation to include a narrow initial portion of a recording and the controlled nature of the read speech task, or the even more controlled task of counting out loud, appear to contradict this design aim. How well AVQI measures obtained from heavily controlled speech material can be transferred to less controlled speech, such as when speaking spontaneously, has not received attention in previous reports. The choice of recommending strict control over the injected material to achieve stability in measurements may be premature, as the review found no investigations into the acoustic measurement robustness of the AVQI, and how it can be enhanced to support assessment of a speaker's voice with ensured internal consistency. The lack of cross-validation procedures in most studies further compounds these reliability concerns, as performance estimates may be inflated due to overfitting to training data.

Lossy compression was used in one study for storing acoustic recordings prior to AVQI measurement. Of importance for further digital health adoption of the AVQI is that the practice and potential impact of lossy compression on the reliability and validity of assessments are thoroughly discussed. Lossy compression of voice signals is known to introduce some variation in measurement outcomes (102–104), but there are currently no recommendations against it in the AVQI literature, and no evaluation of the impact of lossy compression on AVQI measurements. The practice of using lossy compression to save storage space is, however, not found to be widespread, and currently not a substantial threat to the reliable clinical use of the AVQI. With an implementation of AVQI into smartphones being planned (7), the impact of microphone quality and lossy compression (102) may, however, demand formal evaluation to provide well-supported recommendations that ensure a maintained validity in clinical use of AVQI in the future.

#### Conclusion

While the collection of the sustained vowel material for an AVQI is performed in an approximately equivalent manner, there is substantial variance in how much continuous speech is used in validation studies across languages. The speakers are generally asked to read a standard text to elicit the continuous speech material, of which all but the voiced portions of the initial 3 s are excluded when making an AVQI measurement according to the recommended procedure. Whether the AVQI exhibits good internal consistency and can be generalized to all possible measurement results that could have been obtained from a voice, and methods for achieving a robust outcome, demands increased attention.

## Study 2—determination of the read and spontaneous speech length required to achieve internal consistency in AVQI assessments

### Study aim

The results from Study 1 showed that the internal consistency of AVQI, in terms of measurement outcomes being replicable in other portions of speech in the same recording, demands increased attention. Further, transfer from speech with controlled content, predominantly read speech, to spontaneous speech has not been explored. In Study 2, an alternative strategy to achieve robustness by increasing the length of analyzed continuous speech was explored. Utterances of both read and spontaneous speech were generated by concatenation of recorded utterances in the simulation procedure, to support an evaluation of the ability to achieve good internal consistency in both read and spontaneous speech.

### Methods

This study received ethical approval following review by The Swedish Ethical Review Authority (Case number 2022-05630-02).

### Materials

Recordings of sustained vowels, read speech, and spontaneous speech were collected from 54 native speakers of Swedish (22 female, 32 male) using a Zoom H4n Pro recorder (48 kHz sampling rate). The speakers read two standard texts [“A Difficult Case” [Ett svårt fall] and “The Trapetize Artist” [Trapetskonstnären] (105–107)] and held a monologue related to a topic that they were interested in. All sentences in the text readings and spontaneous speech monologues were identified manually, marked for start and end times, and further annotated in terms of the number of words produced in the sentence. The duration in seconds was also noted for each utterance. See Table 1 for a summary of speaker demographics and a presentation of the number of read and spontaneous speech utterances, and the number of words and duration (in number of seconds) of these utterances.

**Table 1:** Demographic information on the participants and an overview of the continuous speech materials identified in the read and spontaneous speech recordings. The results of a statistical test of differences between materials produced by female and male participants are also indicated.

Characteristic	Overall *N* = 54	Female *N* = 22	Male *N* = 32	*p*-value^a^
Particpants				
Speaker age				0.4
Median (Q1, Q3)	66.0 (57.0, 73.0)	62.0 (54.0, 74.0)	67.0 (60.5, 72.5)	
Min; Max	46.0; 78.0	48.0; 78.0	46.0; 78.0	
Read speech				
Number of utterances				0.4
Median (Q1, Q3)	15.5 (12.0, 23.0)	17.0 (11.0, 22.0)	15.5 (12.5, 23.0)	
Min; Max	8.0; 32.0	9.0; 25.0	8.0; 32.0	
Number of words				0.084
Median (Q1, Q3)	7.2 (6.6, 7.9)	7.4 (6.9, 8.4)	7.0 (6.5, 7.6)	
Min; Max	5.1; 10.3	5.1; 10.3	5.2; 9.9	
Duration (s)				0.011
Median (Q1, Q3)	2.7 (2.3, 3.2)	3.0 (2.7, 3.2)	2.6 (2.1, 3.0)	
Min; Max	1.4; 5.9	1.9; 5.9	1.4; 4.7	
Spontaneous speech				
Number of utterances				0.6
Median (Q1, Q3)	16.5 (12.0, 23.0)	16.0 (11.0, 24.0)	16.5 (12.5, 22.5)	
Min; Max	5.0; 37.0	5.0; 28.0	5.0; 37.0	
Number of words				0.4
Median (Q1, Q3)	6.2 (5.5, 7.3)	6.5 (5.5, 7.4)	6.2 (5.6, 7.0)	
Min; Max	4.2; 11.8	4.2; 11.8	4.6; 10.0	
Duration (s)				0.021
Median (Q1, Q3)	2.1 (1.7, 2.5)	2.3 (2.0, 2.7)	1.9 (1.5, 2.3)	
Min; Max	1.1; 4.2	1.1; 4.2	1.3; 3.5	

a Wilcoxon rank sum test of differences between genders.

### Procedure

Each produced utterance was extracted from the recordings and placed in a separate sound file. From the read speech utterances, unique continuous speech materials were constructed by iteratively selecting an increasing number of the utterances at random from the pool of utterances for a speaker. The selected utterances were then concatenated into one continuous speech material. The first set, therefore, contained one randomly selected utterance, the second set was constructed from two randomly selected utterances concatenated together, the third set was the concatenation of three randomly selected utterances, and so on. Finally, the largest continuous speech material was made from all utterances produced in the task by a speaker, concatenated together in order of their random selection. The spontaneous speech materials for each speaker were constructed using the same procedure.

### Acoustic analysis

All read and spontaneous speech materials were combined with a sustained vowel with a 3-second duration, and an AVQI measure was computed. The median values of sub-measures from which the AVQI is computed (CPPS, HNR, LTAS Slope, LTAS Tilt, Shim local, and Shim local DB) were also noted. All acoustic measurements were performed using the official implementation of AVQI v3.01 for Praat using the parselmouth software package (108).

### Statistical analysis

A per-speaker median was calculated for the acoustic measures as an estimate of the true value for the speaker (Table 2). The difference between measures obtained from constructed sets of continuous speech materials and the corresponding speaker median values was analyzed in terms of the observed variability as a function of the length of continuous speech material used. The 0.95 and 1.36 levels of deviation from AVQI medians were used as visual references indicating clinically meaningful worsening or improvement in disability by patients and clinical experts, respectively (4). Separate analyses were performed for read and spontaneous speech, and approximate desirable speech lengths were determined from the visualizations of spread from the speaker median for AVQI or an acoustic subcomponent of AVQI. A good internal consistency in measurement was considered to have been achieved when 1) the constructed continuous speech samples within a length range (5 words or 5 s, respectively) were within subclinical deviation from the speaker median, and 2) the samples' median (the box plot center line) stabilized around the zero line with no notable deviations form that line when larger constructed samples were analyzed. Differences in material lengths produced by female and male speakers, both in terms of the number of words and in the duration of utterances in seconds, were assessed using Wilcoxon rank sum testing (Table 1). Similarly, gender differences in acoustic outcomes (regarding the AVQI and its subcomponents) were assessed using the Wilcoxon rank sum test (Table 2). All statistical testing, data preparation, and visualizations were performed in the R programming language (109).

**Table 2:** Overview of the median AVQI and per speaker median values of all acoustic properties from which the AVQI is computed. The results of a statistical test of differences between acoustic measurements of female and male participants are also presented.

Characteristic	Overall *N* = 54	Female *N* = 22	Male *N* = 32	*p*-value^a^
Speaker medians for acoustic measures				
AVQI				0.6
Median (Q1, Q3)	3.2 (1.9, 4.5)	3.1 (2.4, 4.2)	3.3 (1.7, 4.8)	
Min; Max	1.3; 7.5	1.5; 6.6	1.3; 7.5	
CPPS				>0.9
Median (Q1, Q3)	12.1 (9.9, 14.3)	12.1 (10.7, 14.0)	12.2 (9.6, 14.4)	
Min; Max	6.3; 15.5	7.7; 15.0	6.3; 15.5	
HNR				0.071
Median (Q1, Q3)	13.9 (11.3, 16.9)	14.7 (12.8, 17.8)	13.0 (10.8, 16.7)	
Min; Max	7.1; 19.3	11.0; 19.0	7.1; 19.3	
LTAS Slope				0.005
Median (Q1, Q3)	−21.6 (−24.4, −19.5)	−20.5 (−22.9, −17.9)	−23.0 (−25.3, −20.6)	
Min; Max	−29.5; −9.3	−25.2; −9.3	−29.5; −15.3	
LTAS Tilt				0.13
Median (Q1, Q3)	−11.3 (−11.8, −10.4)	−11.1 (−11.6, −10.0)	−11.6 (−11.9, −10.4)	
Min; Max	−13.2; −8.6	−13.0; −8.6	−13.2; −9.4	
Shim (local)				0.3
Median (Q1, Q3)	8.1 (5.7, 11.5)	7.7 (5.6, 10.7)	9.0 (5.9, 12.6)	
Min; Max	3.0; 17.3	4.3; 14.9	3.0; 17.3	
Shim (local, DB)				0.3
Median (Q1, Q3)	0.8 (0.7, 1.1)	0.8 (0.6, 1.0)	0.9 (0.7, 1.1)	
Min; Max	0.6; 1.5	0.6; 1.3	0.6; 1.5	

a Wilcoxon rank sum test of differences in acoustic outcomes between genders.

### Results

**Figure 1:**
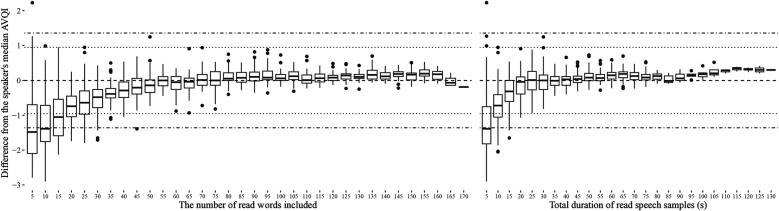
Variation in AVQI measures relative to the speakers' median AVQI as a function of the length of read speech material used. The length of read speech material is displayed in terms of the number of words in the continuous speech (left) and in terms of total speech duration in seconds (right). Horizontal reference lines indicating estimated levels of clinically meaningful change (4) as perceived by experts (dotted lines) and as reported by patients in relation to the perceived level of disability (dashed and dotted lines) are also provided.

**Figure 2:**
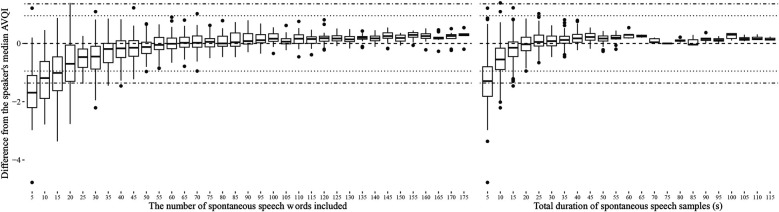
Variation in AVQI measures relative to the speakers' median AVQI as a function of the length of spontaneous speech material used. The length of read speech material is displayed in terms of the number of words in the continuous speech (left) and in terms of total speech duration in seconds (right). Horizontal reference lines indicating estimated levels of clinically meaningful change (4) as perceived by experts (dotted lines) and as reported by patients in relation to the perceived level of disability (dashed and dotted lines) are also provided.

The stability of AVQI as a function of how much continuous speech material it was based on is presented in Figure 1 for read speech and Figure 2 for spontaneous speech. The corresponding displays for CPPS, HNR, LTAS Slope, LTAS Tilt, Shim local, and Shim local DB, from which the AVQI is computed, are presented in Supplementary Materials B. No indications of clinically meaningful differences are, however, provided for these acoustic properties as they have not been determined. The AVQI measure displayed a substantial risk of deviating to a clinically meaningful extent from the speaker's median AVQI if continuous speech of less than 20 words (or 10 s) is used for both the read speech (Figure 1) and continuous speech (Figure 2) materials. Stable AVQI measures were, however, achieved when more materials are added, and once stability was achieved, only small, sub-clinical fluctuations were observed for both continuous speech types.

**Figure 3:**
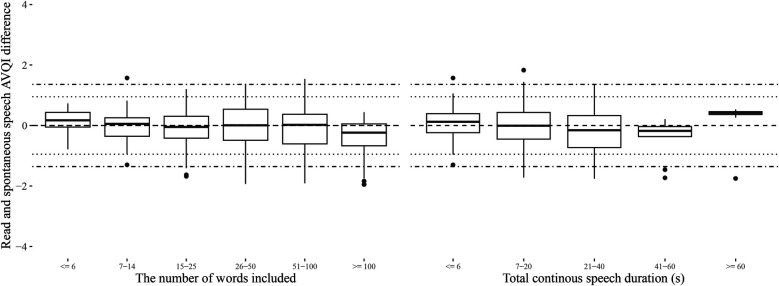
The differences between AVQI obtained from read and spontaneous speech in length (number of words or duration in seconds) categories that approximate the range of alternatives observed in the literature (Supplementary Materials A). Horizontal reference lines indicating estimated levels of clinically meaningful change (4) as perceived by experts (dotted lines) and as reported by patients in relation to the perceived level of disability (dashed and dotted lines) are also provided.

The estimated length of continuous speech materials required to achieve stable measurements of AVQI is summarized in Table 3, along with the corresponding estimates of the acoustic sub-measures from which the AVQI is computed. The LTAS measures are observed to stabilize earlier than all other sub-measures, which stabilize at the approximate length of material where the AVQI measure stabilizes. Differences between AVQI measures obtained from read and spontaneous speech remain within clinically meaningful limits (Figure 3) across approximate continuous speech length ranges found in the literature (Supplementary materials A). An AVQI obtained from read speech samples of sufficient length, conservatively estimated to be greater than 50 words and 20 s, is observed to afford transfer to spontaneous speech within clinically motivated confidence limits.

**Table 3:** The approximate length of continuous speech after which AVQI and its acoustic sub-measures achieved stable levels. The continuous speech lengths are measured in terms of number of words or the total duration (in seconds).

Acoustic property	Continuous speech duration		Number of words	
	Read speech	Spontaneous speech	Read speech	Spontaneous speech
AVQI	>20 s	>20 s	>50	>50
CPPS	>20 s	>15 s	>35	>30
NHR	>20 s	>15 s	>40	>30
LTAS slope	>5 s	>5 s	>5	>10
LTAS tilt	>5 s	>5 s	>5	>10
Shimmer (local)	>20 s	>15 s	>40	>50
Shimmer (local, DB)	>20 s	>15 s	>40	>50

### Discussion

The results indicate that internal consistency in AVQI measurements of a speaker is achieved when approximately 50 words, or approximately 20 s, or more of continuous speech is included. Consistency is achieved at the same approximate continuous speech lengths for both read and spontaneous speech, and the difference between AVQI measures obtained for these two kinds of continuous speech remains subclinical across all relevant length ranges. The results suggest that too short samples produce both a situation where AVQI is highly unstable, which is interpreted as being caused by a high dependence on the exact sentence used, and also may systematically underestimate the AVQI. If a continuous speech of sufficient length is included, however, AVQI results are shown to be relatively unaffected by the exact sentences analyzed. The results further indicate an affordance for transferring conclusions from read speech to continuous speech.

## Overall discussion

The AVQI measure is a digital health assessment procedure for dysphonia severity that has received much recent attention for its detection accuracy, has been most extensively validated across a wide range of languages, and is being evaluated for remote assessment and screening using a mobile phone application. Successful broad adoption of a digital health assessment procedure hinges on the consistency in outcomes being maintained when it transferred to a less controlled setting. The narrative review presented here, however, indicated a need to put additional focus on measurement reliability with regards to the AVQI measure. There is currently no standard for the amount of continuous speech material that should be collected across language-specific validation efforts adopted across languages, and validation and application studies do not always follow the same data collection procedures. Further, the performance of the AVQI has not, in general, been evaluated in a separate validation set, which means that performance estimates are open to being inflated, to an unknown degree, due to overfitting the training data. Overfitting is a serious concern when building automatic assessment techniques, and the severity of the effect of this issue requires immediate attention in relation to all validation studies. It is suggested that AVQI threshold values should be deduced in a training set of 75%–80% of the data that should be selected in a stratified manner with regard to medical condition and speaker age and gender, so that the training and evaluation sets have comparable distributions for these demographic properties. Estimating the sensitivity, specificity, ROC AUC, and other measures should then be done only based on the 20%–25% of the speakers not included in the training set. It is expected that reported performance metrics of AVQI will be reduced as a result of this modified evaluation procedure, but the results will provide a more reliable point of reference for subsequent research in which the AVQI threshold is applied to assess new speakers.

As a secondary consequence of a revised AVQI threshold determination procedure, the only existing recommendation of using the equivalent of 3 s of voiced continuous speech, while not adhered to in all studies, may require revision. Clear measurement guidelines are essential to ensure reliable results, but the guidelines themselves need to be determined by reliable evaluation procedures. In the case of the AVQI recommendation of 3 s of voiced portions of continuous speech (23), the conclusion was the result of an observation of somewhat better performance in a material of this structure compared to, for instance, when an entire standard text was included. The improvement observed using 3 s compared to the whole standard text was, however, not substantial. Since performance metrics were evaluated in the training data, it is further reasonable to assume that they are inflated. The size of the effect of overfitting is not often estimated and reported in studies, but the results from one report (110) indicate an up to 4x improvement in explained variance of a regression model when evaluated within the training data compared to a separate validation data set, which indicates that the implications of overfitting to the training data should not be disregarded. One should, however, not presuppose that the performance of AVQI computed from a 3 s material is reduced to the same degree as when computed from a larger portion of text when assessed in separate evaluation data. In fact, the results presented here suggest that longer materials may provide AVQI measurements that are less sensitive to changes in material. Differences in AVQI performances across training and evaluation data require, however, a separate evaluation and should be the target of further research.

The results suggest that a reliable AVQI for a speaker requires substantially longer recordings of text readings (approximately 50 words or 20 s) than the recommendation and the material used in many validation efforts. While a high level of reliability of AVQI can be achieved by consistent use of materials for its computation, this strategy does not transfer to a high internal consistency of the procedure. Further, it requires consistency use of procedures in validation and application studies, which was not always observed in the narrative review. If consistent use is not achieved across research studies, inconsistent clinical use of the assessment procedure is likely to occur. Therefore, methods for ensuring increased AVQI robustness to speech material differences, such as increasing the length of included speech, appear to provide a better way forward in the adoption of the AVQI in clinical assessment of dysphonia.

The AVQI is an established and widely adopted acoustic assessment method for dysphonia detection and assessment of severity that has been validated in substantial samples of speakers. In a digital health context, an ensured data quality is highlighted as requiring increased attention (111), and the results presented here highlight key areas of improvement for the AVQI procedure which can likely be achieved with relative ease. In most local care contexts, standardized texts longer than the 50 words shown here to provide internal consistency for AVQI are in clinical use in voice-based assessments. Therefore, a simple adjustment of only the acoustic analysis phase may be all that is needed to improve AVQI reliability and generalizability. Estimation of AVQI threshold and evaluation of the performance of the selected threshold in separate sets of speakers is likely to provide more reliable estimates of the assessment accuracy of AVQI. Further, comparisons with patient-reported outcome measures (51, 112) may become more appropriate due to this adjustment. The speaker's gender has been indicated not to influence the AVQI (21, 39, 63) and the effect of speaker age may exist, predominately, when comparing speakers with substantial age differences (75). Nevertheless, age and gender matching as well as an ensured balance in cases and controls are cornerstone improvements on which further development of the measure hinges, and should crucially be included in the design of the coming validation efforts for the AVQI assessment measure.

This study has methodological limitations that should be considered when evaluating the findings. First, the narrative review used only two databases for identifying studies to be included. The databases are large and include both reports published in scientific journals and in scientific reports written by for other audiences. However, studies that would have been of interest to incorporate into the reviewed material may still have been omitted by the methodological decision to include search results from only two databases. Furthermore, the review was conducted by only one person, and instances where another reviewer would have produced a summary of the report with minor variations compared to what is reported here are likely. However, with the purpose of providing a narrative review of a single focused aspect of reports, the impact of both discrepancies between raters' readings of a report, and the failure to include singular reports, is suggested to have a limited impact on the narrative review conclusion of reliability of measurements largely having not been considered with regards to AVQI. A further limitation lies in the relatively small number of speakers from whom material was used in the simulation study. This issue warrants further attention, and the results should be considered for replication in larger speaker samples and other languages before drawing a definitive conclusion on the amount of speech required to provide a reliable AVQI measurement of a speaker's voice.

## Conclusion

The Acoustic Voice Quality Index (AVQI) has received broad adoption in research. However, to ensure reliable application for dysphonia assessment and screening in a digital health context, standardization of the data acquisition procedure towards increased reliability is warranted. The data presented here suggests that text readings longer than 50 words, or longer than 20 s, provide reliable AVQI estimates for a speaker, and further that the results from text readings of this length can be transferred to spontaneous speech. In most contexts in which AVQI has been investigated, the inclusion of the entire reading of the standard text used in clinical practice is likely sufficient to achieve a reliable assessment. It is suggested that methods aimed at ensuring a generalizable estimate of reliability and validity should be given additional focus in the coming research on the AVQI, to support the potential for reliable application of the method in digital health assessments and screening.

## Data Availability

The datasets presented in this article are not readily available because the original sound files cannot be shared due to them being personal identifiable information under national law. Requests to access the datasets should be directed to fredrik.nylen@umu.se.
