# Supplementary material for: On Acoustic Voice Quality Index measurement reliability in digital health applications: a narrative review and empirical evaluation of speech sample length requirements
Source: Front Digit Health. 2025 Nov 24;7:1610353. doi: 10.3389/fdgth.2025.1610353 (PMC12682815; doi:10.3389/fdgth.2025.1610353)
Supplement: Supplementary file 1 [file Datasheet1.docx]

Supplementary Materials A

Supplementary table 1: Information extracted from studies included in the narrative review.

| Language | Year | Study type | Work | Participants | Sustanined vowel length | Type of continuous speech | Continuous speech length | Outcome | Assessment of reliability of acoustic measurements |
| --- | --- | --- | --- | --- | --- | --- | --- | --- | --- |
| American English | 2023 | Validation | Castillo-Allendes et al. (2023) (17) | 197, unbalanced case / control groups, no gender and age matching | Mid 3s portion of prolonged vowels | Read speech | Approximately 3 s of voiced segments | Significant correlation (r=0.72) with perceptual ratings | — |
| American English | 2023 | Validation | Boutsen et al. (2023) (23) | 14, unbalanced case / control groups, no gender and age matching | Mid 3s portion of prolonged vowels | Read speech | 15 words | r=0.61 correlation between AVQI and dysarthria severity | — |
| American English | 2023 | Validation & Application | McAllister et al. (2023) (24) | 250, unbalanced case / control groups, no gender and age matching | Mid 3s portion of prolonged vowel | Read speech | 27 words (34 syllables) | Association between perceptual evaluation and AVQI (r=0.52). LSVT LOUD reduced AVQI | — |
| American English | 2024 | Application | Brenk et al. (2024) (25) | 18 | N/A | Read speech | 29 words, 13.3-16.1 seconds | Reduction in mean AVQI post treament | — |
| American English | 2024 | Application | Stager & Maryn (2024) (26) | 97 | 3 s | Read speech | 12 words (22 syllables) | Significant correlation between AVQI and glottal stop production meassures | — |
| American English | 2024 | Reliability | Yousef & Hunter (2024) (27) | 5 | Mid 3 s portion of prolonged vowels | N/A | N/A | Stable reproduction of HNR, jitter, CPPs, and alpha ratio under reverberation | — |
| American English | 2025 | Validation | Yousef et al. (2025) (28) | 187, unbalanced case / control groups, no gender and age matching | 3 s | Read speech | 51 words | Sensitivity:0.55, Specificity:0.80 | — |
| Australian English | 2012 | Validation | Reynolds et al. (2012) (29) | 26, unbalanced groups, no case / control matching | Mid 3s portion of prolonged vowels | Read speech | Short text | Accuracy: 80.4% | — |
| Australian English | 2018 | Validation | Lee et al. (2018) (30) | 112, unbalanced case / control groups, no gender and age matching | Mid 3s portion of prolonged vowels | Read speech | 34 words | Correlation with perceptual measures 0.21-0.61 | — |
| Bengali | 2022 | Validation | Bhatt et al. (2022) (19) | 14, unbalanced case / control groups, no gender and age matching | 5 s | Read speech | 54 words | Strong correlation between AVQI and perceptual rating (0.98) | — |
| Brazilian Portuguese | 2020 | Reliability | Englert et al. (2020) (31) | 53 | Mid 3s portion of prolonged vowels | Counting and Read speech | Approximately 3 s of voiced segments | Reading text resulted in higher AVQI than the counting task. | — |
| Brazilian Portuguese | 2021 | Validation | Englert et al. (2021) (32) | 150, unbalanced case / control groups, no gender and age matching | Most stable 3 s of prolonged vowels | Read speech | Approximately 3 s (18.8±6.24 syllables) | Sensitivity: 0.79, Specificity: 0.91, ROC AUC: 0.90 | An optimization procedure around 3 s included continuous speech |
| Brazilian Portuguese | 2022 | Application | Englert et al. (2022) (33) | 258 | Mid 3s portion of prolonged vowels | Counting | Approximately 3 s of voiced segments | Accuracy: 0.7-0.88 in predicting degree of deviation from AVQI | — |
| Brazilian Portuguese | 2023 | Validation | Penido & Gama (2023) (34) | 42, unbalanced case / control groups, no gender and age matching | Mid 3s portion of prolonged vowel | Counting | 10 | Sensitivity: 0.51, Specificity: 0.91 | — |
| Brazilian Portuguese | 2024 | Reliability | Martinho & Constantini (2024) (21) | 47 | N/A | Counting and Read speech | 215 | AVQI does not vary significantly due to gender | — |
| Brazilian Portuguese | 2024 | Validation | Rabelo et al. (2024) (35) | 185, unbalanced case / control groups, no gender and age matching | N/A | Counting out loud | 10 words | ROC AUC:0.76 | — |
| Dutch | 2010 | Validation | Maryn et al. (2010) (1) | 251, unbalanced case / control groups, no gender and age matching | Mid 3 s portion of prolonged vowels | Read speech | 12 words (approximately 4 s) | R= 0.67-0.86 between AVQI and perceptual assessment | Cross-validation of correlation measures |
| Dutch | 2010 | Validation | Maryn et al. (2010) (20) | 251, unbalanced groups, no case / control matching | Mid 3 s portion of prolonged vowels | Read speech | 70 words | 63% explained variance in perceptual assessment, Sensitivity: 0.74, Specificity: 0.96. ROC AUC:0.92 | — |
| Dutch | 2015 | Reliability | Maryn & Weenink (2015) (36) | 289 | Mid 3s portion of prolonged vowel | Read speech | 12 words | r=0.98 between versions of AVQI. | — |
| Dutch | 2016 | Validation | Barsties & Maryn (2016) (5) | 1058, unbalanced case / control groups, no gender and age matching | Mid 3s portion of prolonged vowels | Read speech | 25 words | 64% of variance in perceptual rating explained | — |
| Dutch | 2017 | Application | D’haeseleer et al. (2017) (37) | 26 | Mid 3 s portion of prolonged vowels | Read speech | 70 words | No significant effect of theater performance | — |
| Dutch | 2017 | Validation | Latoszek et al. (2017) (38) | 1028, unbalanced case / control groups, no gender and age matching | 3s | Read speech | 24 words | --- | — |
| Dutch | 2019 | Reliability | Batthyany et al. (2019) (39) | 123 | Mid 3s portion of prolonged vowel | Read speech | 12 words | No significant effects of gender | — |
| Dutch | 2021 | Application | Sluis et al. (2021) (40) | 43 | ≥ 3 s | Read speech | 4 s | Post-operative reduction in AVQI to the approximate level of the language specific cutoff | — |
| Dutch | 2022 | Application | D’haeseleer et al. (2022) (41) | 30 | Mid 3 s portion of prolonged vowels | Read speech | 70 words | Good AVQI in performers while clear presence of discomfort | — |
| Dutch/German/French (presumably) | 2015 | Validation | Barsties & Maryn (2015) (42) | 60, unbalanced case / control groups, no gender and age matching | 3s | Read speech | 17 syllables/3 s/23 syllables | Sensitivity = 0.91-1.00, Specificity = 0.86-0.94 | Materials of three different continuous speech lengths included |
| Estonian | 2016 | Validation | Ello et al. (2025) (43) | 98, unbalanced case / control groups, no gender and age matching | N/A | Read speech | 18 words | Sensitivity: 0.82, Specificity:0.95 | — |
| Finnish | 2020 | Validation | Kankare et al. (2020) (44) | 200, unbalanced case / control groups, no gender and age matching | 5 s | Read speech | 92 words | r=0.74 between AVQI and perceptual ratings | — |
| Finnish | 2021 | Validation | Faham et al. (2021) (45) | 128, unbalanced case / control groups, no gender and age matching | Mid 3s portion of prolonged vowels | Read speech | 21 words (33 syllables) | Sensitivity: 0.85, Specificity: 0.81, ROC AUC:0.54 | — |
| Finnish | 2022 | Application | Laukkanen & Rantala (2022) (46) | 104 | Mid 3s portion of prolonged vowels | Read speech | 11 words (31 syllables) | AVQI, CPPs, and HNR was weakly associated with creak. Strain in voice decreased AVQI | — |
| Finnish | 2024 | Application | Convey et al. (2024) (47) | 12 | Mid 3s portion of prolonged vowel | Read speech | 12 words (33 syllables) | Small improvements in AVQI in 50% of participants | — |
| Finnish | 2024 | Validation | Convey et al. (2024) (48) | 17, unbalanced case / control groups, no gender and age matching | Mid 3s portion of prolonged vowels | Read speech | 12 words (33 syllables) | Significant correlation (r=0.50) with perceptual ratings | — |
| Flemish Dutch | 2014 | Application | Maryn et al. (2014) (18) | 50 | Mid 3s portion of prolonged vowels | Read speech | 11 /17 words (Dutch), 15 / 16 words (English), 14 words (German), 14 words (French) | 61-75% explained variance in dysphonia severity | — |
| French | 2020 | Validation | Pommée et al. (2020) (49) | 167, unbalanced case / control groups, no gender and age matching | 5s | Read speech | 32 syllables | Sensitivity: 0.60, Specificity: 1.00 | — |
| French | 2022 | Reliability | Pommée & Morsomme (2022) (50) | 25 | 3s | Read speech | 2 sentences 27 syllables | AVQI was increased in telephone transfer of speech | — |
| French | 2022 | Validation | Pommée et al. (2020) (51) | 78, unbalanced case / control groups, no gender and age matching | Mid 3 s portion of prolonged vowels | Read speech | 27 syllables | Moderate correlation between AVQI and overall VHI and sub-scores | — |
| German | 2012 | Validation | Barsties & Maryn (2012) (52) | 61, unbalanced case / control groups, no gender and age matching | 6 s | Read speech | 14 words | ROC AUC : 0.89 | — |
| German | 2013 | Reliability | Barsties & Maryn (2013) (53) | 43 | Mid 3 s portion of prolonged vowels | Read speech | 12 words | Correlation between test and retest: 0.96 | — |
| German | 2020 | Validation | Latoszek et al. (2020) (54) | 218, unbalanced case / control groups, no gender and age matching | Mid 3s portion of prolonged vowels | Read speech | 3 s /16-112 syllables, standardized around 27 syllables (17 words) | Sensitivity: 0.72, Specificity: 0.90-0.95 | Comparison with hand measured voiced parts within the 3 seconds |
| German | 2020 | Validation | Stappenbeck et al. (2020) (55) | 175, unbalanced case / control groups, no gender and age matching | Mid 3s portion of prolonged vowels | Read speech | 3 s /16-112 syllables, standardized around 27 syllables (17 words) | Sensitivity: 0.23-0.72, Specificity: 0.90-1.00, ROC AUC:0.89-0.90 |  |
| German | 2023 | Reliability | Gierlich & Latoszek (2023) (56) | 39 | Mid 3 s portion of prolonged vowels | Read speech | 17 words (27 syllables) | ICC:0.81 (95% CI 0.64-0.90) for AVQI test-retest | — |
| German | 2024 | Application | Müller et al. (2025) (57) | 11 | Mid 3s portion of prolonged vowel | Read speech | 17 words, mean duration 6.8 s (27 syllables) | STN DBS increased AVQI, particularly when combined with SNr stimulation | — |
| Italian | 2023 | Validation | Fantini et al. (2023) (58) | 150, unbalanced case / control groups, no gender and age matching | Mid 3 s portion of prolonged vowels | Read speech | Approximately 3 s of 5 CAPE-V sentences (55 syllables) | Sensitivity: 0.90, Specificity: 0.92, ROC AUC: 0.92 | An optimization procedure around 3 s included continuous speech |
| Italian | 2024 | Application | Guastamacchia et al. (2024) (59) | 20 | 3 vowels of 3 s of duration each | Read speech | 124 words | Use of CI elevates AVQI to dysphonic levels | — |
| Italian | 2024 | Application | Rosa et al. (2024) (60) | 224 | Mid 3s portion of prolonged vowel | Read speech | 34 words | 50% of patients had preoperative AVQI above threshold, and remained so 3 months post operatively | — |
| Japanese | 2017 | Validation | Hosokawa et al. (2017) (61) | 336, unbalanced case / control groups, no gender and age matching | 3 s | Read speech | 31 words (65 syllables) | Sensitivity: 0.73, Specificity: 0.95 | — |
| Japanese | 2024 | Validation | Hosokawa et al. (2024) (4) | 110, unbalanced case / control groups, no gender and age matching | Mid 3 s portion of prolonged vowels | Read speech | 13 words | Sensitivity: 0.85-0.87, Specificity: 0.84-0.89, ROC AUC: 0.90-0.95 | — |
| Kannada | 2021 | Validation | Pebbili et al. (2021) (62) | 90, unbalanced case / control groups, no gender and age matching | Most stable 3 s of prolonged vowels | Read speech | 6 words | Moderate correlation (r=0.67) between AVQI and overall VHI and sub-scores | — |
| Kannada | 2022 | Reliability | Shabnam & Pushpavathi (2022) (63) | 138 | 3 s in the most stable portion of the prolonged vowel | Read speech | 6 words | Overall AVQI score was not affected by speaker gender. | — |
| Kannada | 2024 | Application | Nanjundaswamy & Jayakumar (2024) (64) | 20 | Mid 3s portion of prolonged vowels | Read speech | 3 sentences | Eclectic voice therapy improved AVQI in individuals with hyper functional voice disorder | — |
| Kannada | 2024 | Validation | Shabnam et al. (2024) (65) | 134, unbalanced case / control groups, no gender and age matching | Mid 3s portion of prolonged vowel | Read speech | 6 words | Sensitivity: 0.84-1.00, Specificity: 0.83-1.00, ROC AUC: 0.75-0.89 | — |
| Kannada | 2025 | Validation | Shabnam et al. (2025) (66) | 163, unbalanced case / control groups, no gender and age matching | > 5s | Read speech | 5 sentences, 9-12 words per sentence | Sensitivity: 84.4-100.0%, Specificity: 83.1-100.0% | — |
| Kannada, Tamil, | 2022 | Application | Jayakumar et al. (2022) (3) | 200 | Mid 3s portion of prolonged vowels | Read speech | N/A | AVQI differences between pediatric and adult participants | — |
| Korean | 2016 | Validation | Maryn et al. (2016) (67) | 60, unbalanced case / control groups, no gender and age matching | Mid 3 s portion of prolonged vowels | Read speech | 10 words (25 syllables) | r=0.911 between AVQI and perceptual evaluation | — |
| Korean | 2019 | Validation | Kim et al. (2019) (68) | 1524, unbalanced case / control groups, no gender and age matching | Mid 2s portion ofthe most stable prolonged vowel (3 attempts) | Read speech | 2 sentences (26 syllables) | Sensitivity: 0.90, Specificity: 0.97,ROC AUC: 0.970-0.977 | — |
| Korean | 2020 | Validation | Kim et al. (2020) (69) | 2257, unbalanced case / control groups, no gender and age matching | Mid 2 s portion of prolonged vowels | Read speech | 10 words (25 syllables) | Sensitivity: 0.90, Specificity: 0.94, ROC AUC: 0.96 | — |
| Korean | 2021 | Validation | Kim et al. (2021) (70) | 222, unbalanced case / control groups, no gender and age matching | 3 s | Read speech | 24 words (70 syllables), reduced to around 3 s, around 23 syllables | Sensitivity: 0.90, Specificity: 0.89, ROC AUC: 0.96 | — |
| Korean | 2024 | Validation | Lee & Kim (2024) (71) | 161, unbalanced case / control groups, no gender and age matching | Mid 3 s portion of the most stable version out of 5 prolonged vowels | Read speech | 10 words (25 syllables) | 67% variance explained in perceptual evaluation. | — |
| Korean | 2025 | Validation | Kim et al. (2025) (72) | 177, unbalanced case / control groups, no gender and age matching | 3 s | Read speech | 25 syllables | ROC AUC ≥0.73 | — |
| Lithuanian | 2017 | Validation | Uloza et al. (2017) (73) | 184, unbalanced case / control groups, no gender and age matching | Mid 3s portion of a 5 s vowel | Read speech | 4 words | Sensitivity = 0.840 and Specificity = 0.922 | — |
| Lithuanian | 2018 | Validation | Uloza et al. (2018) (74) | 264, unbalanced case / control groups, no gender and age matching | Mid 3s portion of prolonged vowel | Read speech | 4 words | Sensitivity: 0.78, Specificity: 0.92, ROC AUC:0.92-0.95 in predicting perceptual evaluations | — |
| Lithuanian | 2019 | Reliability | Latoszek, Ulozaitė-Stanienė, Maryn et al. (2019) (75) | 123 | 3 s | Read speech | 4 words | Negligable effect of age | — |
| Lithuanian | 2019 | Validation | Latoszek, Ulozaitė-Stanienė, Petrauskas et al. (2019) (2) | 264, unbalanced case / control groups, no gender and age matching | Mid 3s portion of prolonged vowel | Read speech | 4 words | 79% classification accuracy | — |
| Lithuanian | 2019 | Validation & Reliability | Ulozaite-Staniene et al. (2019) (76) | 183, unbalanced case / control groups, no gender and age matching | Mid 3s portion of prolonged vowel | Read speech | 4 words | Sensitivity: 0.70, Specificity: 0.86, ROC AUC:0.82-0.85. A minimal cutoff difference (0.01) between recording devices was highlighted. | Recording devices were compared based on simultaneously obtained recordings, and identical materials. |
| Lithuanian | 2023 | Application | Maskeliūnas et al. (2023) (77) | 134 | N/A | Read speech | 4 words | ROC AUC ≥ 0.8 for assessments across devices | Optimization of AVQI computations and validation in separate data |
| Lithuanian | 2023 | Reliability | Uloza, Ulozaitė-Stanienė, Petrauskas & Kregždytė (2023) (78) | 183 | Mid 3s portion of prolonged vowel | Read speech | 4 words | Good agreement between AVQI obtained from studio and smartphone voice recordings | Comparisons made from simultaneously obtained voice recordings. |
| Lithuanian | 2023 | Validation | Uloza, Ulozaitė-Stanienė, Petrauskas, Pribuišis, Ulozienė et al. (2023) (79) | 135, unbalanced case / control groups, no gender and age matching | Mid 3 s portion of prolonged vowels | Read speech | 4 words | Sensitivity: 0.94–0.95, Specificity: 0.93–0.96, ROC AUC: 0.97 | — |
| Lithuanian | 2023 | Validation | Uloza, Ulozaitė-Stanienė, Petrauskas, Pribuišis, Blažauskas et al. (2023) (80) | 135, unbalanced case / control groups, no gender and age matching | Mid 3s portion of prolonged vowel | Read speech | N/A | ICC: 0.98 between recording devices. ROC AUC: 0.83-0.86 | — |
| Lithuanian | 2023 | Validation | Uloza et al. (2024) (81) | 129, unbalanced case / control groups, no gender and age matching | 3 s | Read speech | 4 words | Sensitivity: 0.71, Specificity: 0.94, ROC AUC:0.87 | — |
| Malayalam & Kannada | 2024 | Validation | Jayakumar et al. (2024) (82) | 160, unbalanced case / control groups, no gender and age matching | ≥3 s | Read speech | Approximately 3 s of voiced segments | Sensitivity: 0.65, Specificity: 0.95, ROC AUC:0.86 | — |
| Persian | 2024 | Application | Rahimifar et al. (2024) (83) | 60 | Mid 3s portion of prolonged vowel | Read speech | Approximately 3 s of voiced segments | Elevated AVQI (mean difference: 2.79) in PD speakers compared to controls | — |
| Persian | 2024 | Validation | Asiaee et al. (2024) (84) | 221, unbalanced case / control groups, no gender and age matching | 3 s | Read speech | 16 words (36 syllables) | Sensitivity: 0.89, Specificity: 0.54, ROC AUC: 0.77 | — |
| Spanish | 2017 | Validation | Núñez-Batalla et al. (2017) (85) | 112, unbalanced case / control groups, no gender and age matching | 3 s | Read speech | 37 words | 46% of variance in perceptual rating explained | — |
| Spanish | 2018 | Validation | Hernández et al. (2018) (86) | 183, unbalanced case / control groups, no gender and age matching | Mid 3s portion of prolonged vowels | Read speech | 4 randomized sentences (45 syllables) | Sensitivity: 0.75, Specificity: 0.95. ROC AUC: 0.91 | — |
| Spanish | 2022 | Application | Gómez et al. (2022) (87) | 112 | Mid 3s portion of prolonged vowels | Read speech | 4 randomized sentences (45 syllables) | r=0.38 correlation between AVQI and biggest nodule | — |
| Spanish | 2024 | Application | Moya-Galé et al. (2024) (88) | 65 | Mid 3s portion of prolonged vowels | Read speech | 27 words (43 syllables) | Improvement of LSVT LOUD | — |
| Spanish | 2024 | Validation | Pah et al. (2024) (89) | 100, unbalanced case / control groups, no gender and age matching | 3 s | Read speech | 22 words (33 sylables) | Sensitivity: 0.41-0.59, Specificity: 0.61-0.92 | Iterative concatenation of sustained vowel samples up to 6 s, 3 s was then used |
| Swedish | 2022 | Application | Steurer et al. (2022) (90) | 83 | Mid 3s portion of prolonged vowels | Read speech | 22 words (45 syllables) | No treatment effect in AVQI | — |
| Swedish | 2024 | Application | Steurer et al. (2024) (91) | 95 | Mid 3s portion of prolonged vowels | Read speech | 22 words (45 syllables) | 39% of participants showed clinically meaningful decrease poste treatment | ICC: 0.97 computed by re-measuring 20% of the samples |
| Turkish | 2020 | Application | Sagiroglu & Kurtul (2020) (92) | 29 | ≥ 3 s | Read speech | 200 words | 0.55 AVQI increase post radiotherapy, remediation to previous levels 6 months post treatment | — |
| Turkish | 2022 | Validation | Yeşilli-Puzella et al. (2022) (93) | 255, unbalanced case / control groups, no gender and age matching | 3 s | Read speech | 9 words (25 syllables) | Sensitivity: 0.75, Specificity: 0.92, ROC AUC: 0.90 | Retest-reliability assessed in 15% of samples 15 minutes after the first evaluation. ICC=0.99 |
| Turkish | 2024 | Validation | Yeşilli-Puzella et al. (2024) (94) | 255, unbalanced case / control groups, no gender and age matching | 3 s | Read speech | 36 syllables | Sensitivity : 80%, Specificity: 94%,ROC AUC= 0.90 | — |
| Turkish | 2025 | Validation | Gölaç et al. (2025) (83) | 179, unbalanced case / control groups, no gender and age matching | Mid 3 s portion of prolonged vowels | Read speech | 3 s | Significantly different AVQI for dysphonia grades 0-3 | — |
