# Supplementary material for: On Acoustic Voice Quality Index measurement reliability in digital health applications: a narrative review and empirical evaluation of speech sample length requirements
Source: Front Digit Health. 2025 Nov 24;7:1610353. doi: 10.3389/fdgth.2025.1610353 (PMC12682815; doi:10.3389/fdgth.2025.1610353)
Supplement: Supplementary file 2 [file Datasheet2.docx]

Supplementary materials B

Supplementary Figure 1: Variation in the sub-measures from which AVQI is computed relative to the speakers’ median (See Table 2) as a function of the number of read speech words that were included.

Supplementary Figure 2: Variation in the sub-measures from which AVQI is computed relative to the speakers’ median (See Table 2) as a function of the duration of read speech included.

Supplementary Figure 4: Variation in the sub-measures from which AVQI is computed relative to the speakers’ median (See Table 2) as a function of the duration of spontaneous speech included.

Supplementary Figure 4: Variation in the sub-measures from which AVQI is computed relative to the speakers’ median (See Table 2) as a function of the number of read speech words that were included.
